# Morphological traits, photosynthetic pigments and phenolic metabolism in globe artichoke (*Cynara scolymus* L.) genotypes

**DOI:** 10.7717/peerj.21771

**Published:** 2026-10-01

**Authors:** İbrahim Sönmez

**Affiliations:** Vegetable Production and Breeding, Atatürk Central Horticultural Research Institute, Yalova, Turkey

**Keywords:** *Cynara scolymus*, Phenolic compounds, Antioxidant activity, Chlorophyll, Genotype variation, Multivariate analysis

## Abstract

Globe artichoke (*Cynara scolymus* L.) is a phenolic-rich vegetable with high nutritional and antioxidant value; however, integrated evaluations of morphological traits, photosynthetic pigments, and phenolic metabolism among local genotypes remain limited. This study compared six genotypes collected from different locations in the Marmara Region of Turkey and grown under uniform environmental conditions. Yield-related traits, photosynthetic pigments (chlorophyll a, chlorophyll b, total chlorophyll, and carotenoids), total phenolics, flavonoids, and antioxidant capacity were evaluated using the 2,2-diphenyl-1-picrylhydrazyl (DPPH) radical scavenging and cupric reducing antioxidant capacity (CUPRAC) assays, while individual phenolic compounds were identified and quantified by high-performance liquid chromatography coupled with diode-array detection (HPLC-DAD). Significant genotypic variation was observed across all parameters. Head weight ranged from 223.19 to 327.65 g, with the highest value recorded in Hamitler. Bilecik exhibited the highest chlorophyll a and total chlorophyll content, whereas Pamukova showed the highest phenolic content and antioxidant capacity. Chlorogenic acid was identified as the predominant phenolic compound. Principal component analysis revealed clear genotype separation mainly driven by phenolic composition and antioxidant activity. These findings demonstrate substantial physiological and biochemical diversity among globe artichoke genotypes and highlight promising materials for breeding programmes aimed at improving both yield and functional quality.

## Introduction

Globe artichoke (*Cynara scolymus* L.) is a perennial species of the *Asteraceae* family widely cultivated in Mediterranean environments for its edible immature inflorescences. In addition to its agronomic importance, artichoke is recognised as a functional food due to its high content of phenolic compounds and associated antioxidant properties (Ceccarelli et al., 2010; Kılıçtaş, Özlü & Garipoğlu, 2020). These bioactive constituents have been linked to various health-promoting effects, including hepatoprotective, cardioprotective, and anti-inflammatory activities (Miraj & Kiani, 2016; Salekzamani, Ebrahimi-Mameghani & Rezazadeh, 2019).

The phytochemical composition of artichoke is largely characterised by caffeoylquinic acid derivatives and flavonoid glycosides (Negro et al., 2012; Pandino, Lombardo & Mauromicale, 2013; Rocchetti et al., 2020). Among these, chlorogenic acid and cynarin are considered key phenolic compounds contributing to antioxidant capacity and are frequently used as biochemical markers in genotype evaluation (Wang et al., 2003; Lombardo et al., 2012; Bonasia et al., 2023). Other compounds such as luteolin derivatives, rhoifolin, and syringin also contribute to the biochemical diversity of artichoke tissues, with their accumulation strongly influenced by genetic background and environmental conditions (Lombardo et al., 2012; Liava et al., 2021; Lombardo et al., 2022).

Antioxidant capacity in artichoke is closely associated with total phenolic and flavonoid content and is commonly evaluated using assays such as DPPH and CUPRAC (Apak et al., 2004; Dabbou et al., 2017; Kayahan & Saloglu, 2020). Previous studies have demonstrated positive correlations between phenolic accumulation and antioxidant activity, although the contribution of individual compounds may vary among genotypes (Petropoulos et al., 2018; Luca et al., 2022; Bonasia et al., 2023).

In addition to secondary metabolites, photosynthetic pigments such as chlorophylls and carotenoids play a critical role in plant physiological performance and may be linked to metabolic processes involved in secondary metabolite biosynthesis (Lichtenthaler & Wellburn, 1983; Liava et al., 2021). Variations in pigment composition can therefore reflect differences in physiological status and adaptive capacity among genotypes (Liava et al., 2021; Rau et al., 2022).

Although numerous studies have examined individual aspects of artichoke physiology or phytochemistry, integrated assessments combining morphological traits, photosynthetic pigments, antioxidant activity, and detailed phenolic profiling across local genotypes remain limited (Ciancolini et al., 2012; Mauro et al., 2012; Alicandri et al., 2023). Therefore, the objective of this study was to evaluate the morphological performance, pigment composition, antioxidant capacity, and phenolic metabolism of globe artichoke genotypes grown under uniform environmental conditions, and to identify genotypes with superior agronomic and functional characteristics.

## Materials and Methods

### Plant material and experimental site

Six globe artichoke (*Cynara scolymus* L.) genotypes were collected from different locations in the Marmara Region of Türkiye (Balıkesir, Bilecik, Bursa, Hamitler, Pamukova, and Taraklı). No specific field permit was required for the collection of the six globe artichoke (*Cynara scolymus* L.) genotypes used in this study, as the plant materials were obtained from cultivated/local plant materials and did not involve protected, endangered, or restricted species. The experiment was conducted at the Atatürk Central Horticultural Research Institute (Yalova, Türkiye) during the 2022 and 2023 growing seasons.

The trial was established in a randomised complete block design with four replications, each consisting of 20 plants per genotype. For yield-related morphological traits, measurements were taken from randomly selected representative plants within each replication, while plant samples used for pigment, phenolic, and antioxidant analyses were collected as subsamples from each replication. Plants were grown under uniform agronomic conditions, and irrigation and fertilisation were applied *via* a fertigation system to minimise environmental variability.

### Soil sampling and analysis

Soil samples were collected prior to the establishment of the experiment from two depths (0–20 cm and 20–40 cm) using a stainless-steel auger following standard procedures (Jackson, 1962). Samples were air-dried, sieved, and prepared for analysis according to Kacar (1994). Soil texture was determined by the Bouyoucos hydrometer method (Bouyoucos, 1951). Soil pH and electrical conductivity were measured in saturated soil paste using a glass-electrode pH meter and an electrical conductivity meter, respectively (Tüzüner, 1990). Lime content was determined volumetrically with a Scheibler calcimeter (Hızalan & Ünal, 1966), and organic matter content was determined using the modified Walkley–Black method (Jackson, 1962).

Exchangeable potassium (K), calcium (Ca), and magnesium (Mg) were extracted using 1 N ammonium acetate (pH 7.0) and quantified by ICP-OES (Cottenie, 1980). Available phosphorus (P) was determined by the sodium bicarbonate extraction method (Olsen et al., 1954). Available micronutrients (Fe, Cu, Zn, and Mn) were extracted with DTPA solution (pH 7.3) and determined by ICP-OES according to Lindsay & Norvell (1978).

### Yield and yield-related traits

Agronomic performance was evaluated at commercial harvest maturity. Head weight, head diameter, head height, and number of heads per plant were recorded from randomly selected plants within each replication. Measurements were expressed on a fresh weight basis.

### Determination of photosynthetic pigments

Artichoke leaf samples were dried and ground into fine powder prior to analysis. A 0.2 g sample was extracted with 10 mL methanol in a screw-cap tube. The mixture was shaken for 4 h and centrifuged at 4,000 rpm for 10 min. Absorbance of the supernatant was measured between 400 and 700 nm using a UV–VIS spectrophotometer.

Chlorophyll a, chlorophyll b, total chlorophyll, and total carotenoid contents were calculated according to Lichtenthaler & Wellburn (1983) using absorbance values at 645, 662, 663 and 470 nm. Pigment concentrations were expressed as mg g^−^^1^ dry weight (DW). The formulas used for methanol extraction were as follows:

(1) \begin{eqnarray*}\mathrm{Chla}~({\text{mg g}}^{-1}~\mathrm{DW})=12.70{A}^{662}-2.69{A}^{645}\end{eqnarray*}

(2) \begin{eqnarray*}\mathrm{Chlb}~({\text{mg g}}^{-1}~\mathrm{DW})=22.90{A}^{645}-4.68{A}^{662}\end{eqnarray*}

(3) \begin{eqnarray*}\mathrm{TChl}~({\text{mg g}}^{-1}~\mathrm{DW})=20.21{A}^{645}+8.02{A}^{663}\end{eqnarray*}

(4) \begin{eqnarray*}\mathrm{TCar}~({\text{mg g}}^{-1}\mathrm{DW})=(1000{A}^{470}-2.27\mathrm{Chl}a-81.4\mathrm{Chl}b)/227.\end{eqnarray*}

### Sample preparation for phenolic and antioxidant analyses

Artichoke samples were dried and ground into powder. For phenolic and antioxidant analyses, 3 g of sample was homogenized with 25 mL methanol and stored at 4 °C overnight. Extracts were centrifuged at 10,000 rpm for 20 min and the supernatants were collected in amber vials and stored at −20 °C until analysis (Stumpf et al., 2019).

### Total phenolic content determination

Total phenolic content (TPC) was determined using the Folin–Ciocalteu method. Briefly, 150 µL extract was mixed with 2,400 µL distilled water and 150 µL Folin–Ciocalteu reagent (0.25 N). After 4 min, 300 µL of 1 N sodium carbonate (Na_2_CO_3_) was added and the mixture was incubated at room temperature for 2 h. Absorbance was measured at 725 nm. Results were expressed as mg gallic acid equivalents (GAE) per 100 g DW using a gallic acid calibration curve (Stumpf et al., 2019).

### Antioxidant activity assays

#### DPPH radical scavenging activity

For the DPPH assay, a working DPPH solution (absorbance 1.1 ± 0.02 at 515 nm) was prepared from a stock solution (0.12 mg DPPH in 50 mL methanol). Extracts (150 µL) were mixed with 2,850 µL DPPH solution and incubated in the dark for 24 h. Absorbance was measured at 515 nm and results were expressed as mg Trolox equivalents (TE) per 100 g DW (Stumpf et al., 2019).

#### CUPRAC assay

The CUPRAC assay was conducted according to Apak et al. (2004). Briefly, 100 µL extract was mixed with one mL each of 10 mM CuCl_2_, 7.5 mM neocuproine, and 1 M ammonium acetate buffer (pH 7.0). Distilled water was added to obtain a final volume of 4.1 mL. After incubation for 60 min at room temperature, absorbance was measured at 450 nm and results were expressed as mg TE per 100 g DW.

#### Bioactive phenolic compound analysis by HPLC

For phenolic profiling, artichoke tissues were homogenized in liquid nitrogen and extracted with ethanol–water (50:50, v/v) at 70 °C for 1 h in a shaking water bath. Extracts were filtered and ethanol was removed under reduced pressure using a rotary evaporator. The remaining aqueous phase was stored at −18 °C until analysis.

Phenolic compounds were analyzed using an HPLC system (HPLC 1100; Hewlett-Packard, Germany) equipped with a diode array detector (DAD). Separation was performed on a Phenomenex Prodigy ODS3 column (150 × 4.6 mm, 2.6 µm). Detection wavelengths were 320 and 335 nm. The mobile phase consisted of solvent A (0.2% phosphoric acid in water) and solvent B (acetonitrile) with a flow rate of 0.6 mL min^−^^1^ and an injection volume of 10 µL. Column temperature was maintained at 27 °C. Phenolic compounds were identified and quantified using external calibration curves prepared from standards including chlorogenic acid, cynarin, luteolin-7-glucoside, luteolin, syringin, and rhoifolin (Wang et al., 2003).

### Statistical analysis

Data obtained from the 2022 and 2023 growing seasons were first subjected to preliminary analyses to evaluate year effects and year × genotype interactions. In the statistical model, genotype was considered as a fixed effect, while year and block/replication effects were treated as random effects. Since the year effect and year × genotype interaction were not statistically significant, data were pooled across years and analysed jointly for genotype comparisons. Analysis of variance (ANOVA) was performed according to a randomised complete block design. Prior to ANOVA, model assumptions were evaluated using residual diagnostics, including visual inspection of residual plots and normal probability plots. These diagnostic evaluations indicated no major deviations from normality or variance homogeneity; therefore, no data transformation was required. Mean comparisons were conducted using Tukey’s multiple comparison test. Differences at the *p* < 0.05 level were considered statistically significant. Statistical analyses were performed using JMP Pro 13 software, following the procedures described by Kalaycı (2005).

## Results

### Soil characteristics of the experimental site

The soil properties of the experimental area were relatively uniform across the two sampling depths (0–20 cm and 20–40 cm), indicating limited spatial variability within the field (Table 1). The soil exhibited a loamy texture with a slightly alkaline reaction and moderate calcium carbonate content. Organic matter content was low in both layers, while available phosphorus decreased with increasing soil depth. Exchangeable nutrients such as calcium and iron were present at adequate levels. Given the uniform soil characteristics and controlled agronomic practices applied throughout the experiment, environmental variation was minimised, allowing for a clearer assessment of genotype-dependent differences.

### Variation in morphological traits among globe artichoke genotypes

Significant differences were observed among globe artichoke genotypes for all evaluated morphological traits (Table 2). Head weight varied between 223.19 and 327.65 g, with the highest value recorded in Hamitler, indicating its superior yield potential. Balıkesir, Bilecik, and Pamukova exhibited intermediate head weights, whereas Bursa and Taraklı showed comparatively lower values.

**Table 1:** Selected soil characteristics (0–20 and 20–40 cm) of the experimental site (texture, EC, pH, CaCO_3_, organic matter, macro- and micro-elements).

Depth (CM)	% Saturation	Electrical Conductivity (EC) µS cm^−1^	pH	CaCO_3_ (%)	Organic Matter (%)	Total Nitrogen (N) (%)	Available Phosphorus (P) (mg kg ^ −1^)	Available Potassium (K) (mg kg ^ −1^)
0–20	46	404	7.78	3.48	1.59	0.103	16.2	293
20–40	46	421	7.75	3.68	1.45	0.094	8.79	185

Depth (CM)	Available Calcium (Ca) (mg kg ^ −1^)	Available Magnesium (Mg) (mg kg ^ −1^)	Available Iron (Fe) (mg kg ^ −1^)	Available Manganese (Mn) (mg kg ^ −1^)	Available Zinc (Zn) (mg kg ^ −1^)	Available Copper (Cu) (mg kg ^ −1^)
0–20	7,330	398	19.84	18.47	1.01	2.85
20–40	7,780	265	20.48	19.54	1.09	2.63

**Table 2:** Morphological traits of globe artichoke genotypes.

Genotypes	Head Weight (g)			Head Diameter (cm)		Head Height (cm)		Head number Per Plant	
Balıkesir	296.29	± 17.68	ab	9.13	± 0.25	8.67	± 0.07	2.61	± 0.40
Bilecik	223.19	± 14.14	b	8.17	± 0.26	7.99	± 0.24	1.63	± 0.24
Bursa	274.84	± 83.07	ab	8.78	± 2.55	8.36	± 2.18	2.82	± 0.34
Hamitler	327.65	± 30.01	a	9.30	± 0.44	8.73	± 0.28	1.63	± 0.13
Pamukova	264.84	± 22.37	ab	8.58	± 0.40	8.14	± 0.40	2.45	± 0.28
Taraklı	230.82	± 22.17	b	8.25	± 0.30	8.01	± 0.28	2.58	± 0.22
CV (%)	12.83			7.52		6.34		4.02	

**Notes.**

Values are expressed as mean ± SE. Different letters within each column indicate significant differences among genotypes according to Tukey’s test (*P* ≤ 0.05).

Head diameter and head height displayed trends similar to head weight, suggesting coordinated development of the edible head structure. Genotypes with higher head weight generally also exhibited larger head dimensions, indicating a positive association among these yield-related traits. The number of heads per plant also varied among genotypes, although this parameter showed less pronounced differences compared to head size characteristics.

### Photosynthetic pigment composition among globe artichoke genotypes

Photosynthetic pigment analysis revealed clear and consistent variation among genotypes (Table 3). Chlorophyll a content ranged from 1.88 to 2.56 mg g^−^^1^ DW, with the highest value observed in Bilecik, followed by Pamukova. The lowest chlorophyll a content was recorded in Hamitler.

**Table 3:** Chlorophyll a, chlorophyll b, total chlorophyll (a+b), and total carotenoid contents of globe artichoke genotypes. Values are expressed as mean concentrations (mg g^−^^1^ DW).

Genotypes	Chlorophyll a			Chlorophyll b			Total Chlorophyll			Total carotenoids		
Balıkesir	1.94	bc	± 0.041	0.84	b	± 0.015	2.78	bc	± 0.035	0.26	b	± 0.019
Bilecik	2.56	a	± 0.067	1.18	a	± 0.015	3.74	a	± 0.078	0.33	a	± 0.012
Bursa	2.04	b	± 0.041	0.88	b	± 0.020	2.92	b	± 0.021	0.34	a	± 0.009
Hamitler	1.80	c	± 0.047	0.81	b	± 0.025	2.61	cd	± 0.035	0.23	b	± 0.015
Pamukova	2.40	a	± 0.015	1.16	a	± 0.049	3.55	a	± 0.064	0.35	a	± 0.009
Taraklı	1.57	d	± 0.033	0.85	b	± 0.044	2.41	d	± 0.012	0.20	b	± 0.012
CV (%)	3.65			5.66			2.70			7.79		

**Notes.**

Values are expressed as mean ± SE. Different letters within each column indicate significant differences among genotypes according to Tukey’s test (*P* ≤ 0.05).

Chlorophyll b and total chlorophyll contents followed patterns similar to chlorophyll a, indicating consistency in pigment composition among genotypes. Total chlorophyll values ranged from 2.84 to 3.74 mg g^−^^1^ DW, further highlighting genotypic differences in photosynthetic capacity.

Carotenoid concentrations also showed significant variation, with Pamukova, Bilecik, and Bursa exhibiting higher levels compared to the other genotypes. In contrast, Taraklı  and Hamitler displayed lower carotenoid accumulation. These differences indicate variability in both light-harvesting pigments and photoprotective compounds among the evaluated populations.

### Phenolic content, flavonoids, and antioxidant activity

Substantial variation was observed among genotypes in total phenolic content, total flavonoids, and antioxidant capacity (Fig. 1). Total phenolic content ranged from 67.50 to 202.00 mg 100 g^−^^1^ DW, with the highest values recorded in Pamukova and Hamitler, whereas Bilecik exhibited the lowest phenolic accumulation.

Total flavonoid content followed a similar trend, although the differences among genotypes were less pronounced. Antioxidant capacity, evaluated using DPPH and CUPRAC assays, showed strong genotypic variation. Pamukova and Balıkesir exhibited the highest DPPH radical scavenging activity, while CUPRAC results confirmed that Pamukova had the highest antioxidant capacity among all genotypes. In contrast, Bilecik and Taraklı showed comparatively lower antioxidant performance.

**Figure 1:**
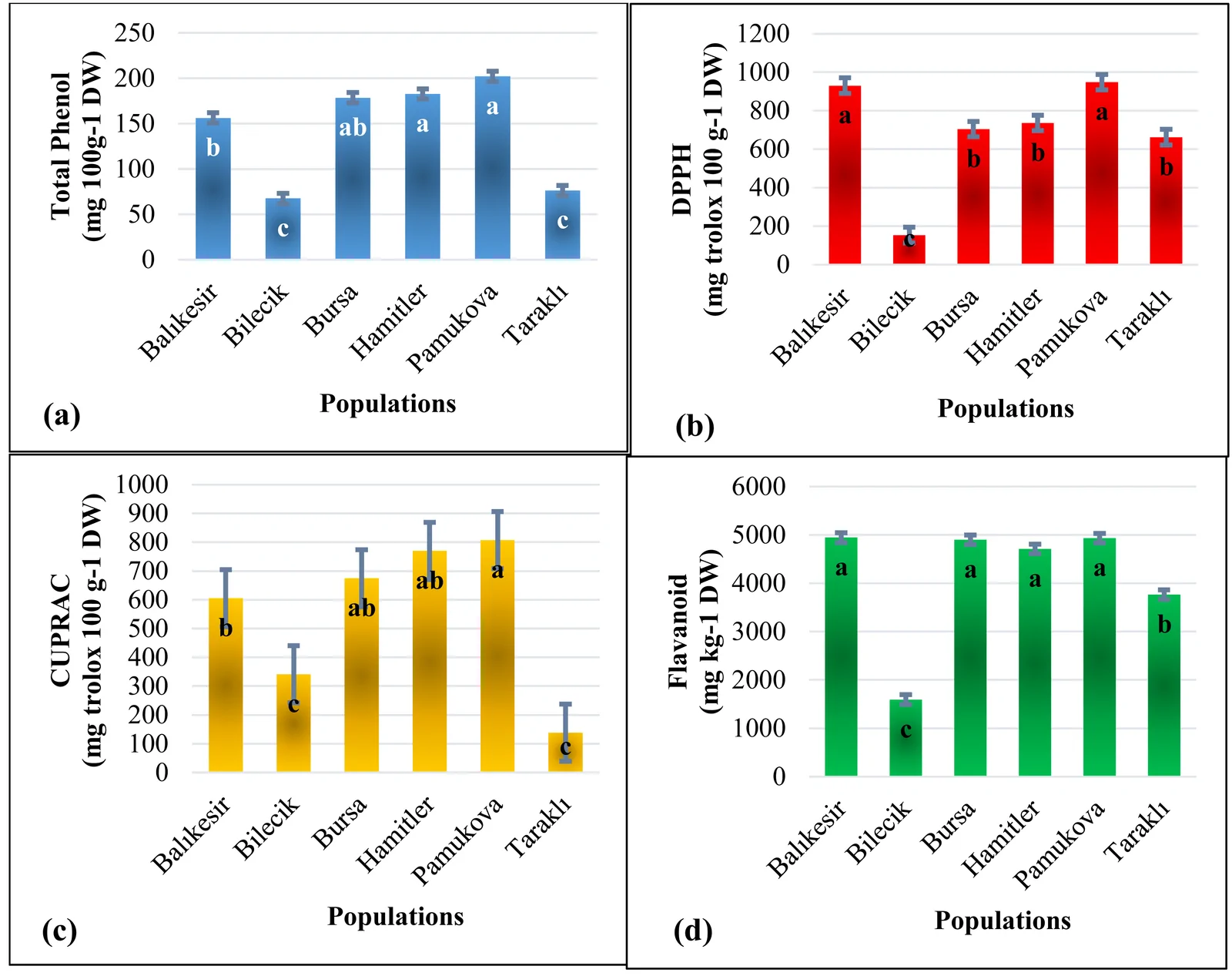
(A–D) Total phenolic content, total flavonoid content, and antioxidant activity of globe artichoke genotypes determined by DPPH and CUPRAC assays.

### Phenolic compounds

The analysis of individual phenolic compounds by HPLC–DAD revealed clear genotype-dependent differences (Fig. 2). Chlorogenic acid was identified as the predominant compound across all genotypes and exhibited the widest range of variation. The highest concentrations were observed in Pamukova, whereas lower levels were recorded in Bilecik.

**Figure 2:**
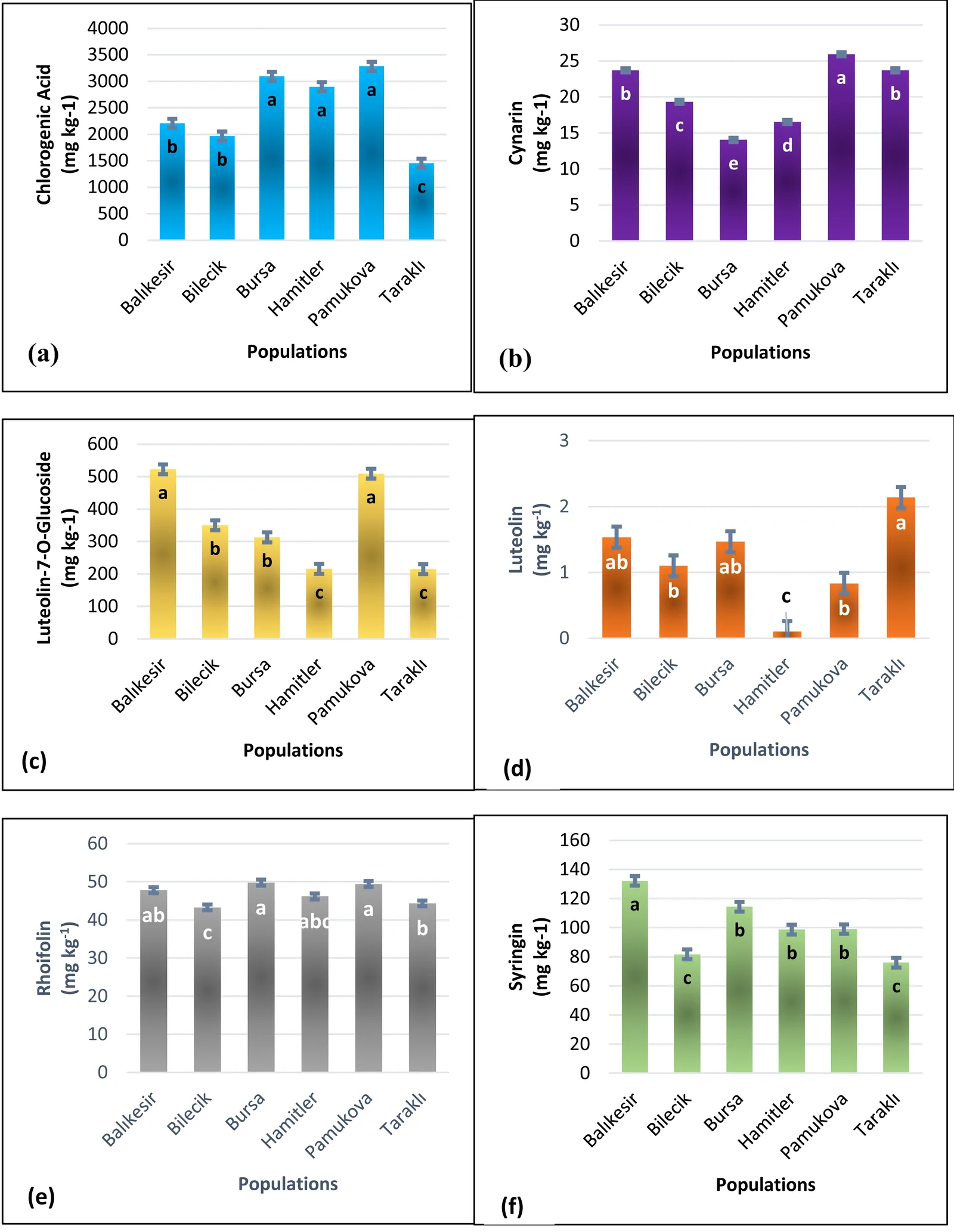
(A–F) Variation in individual phenolic compounds of globe artichoke genotypes determined by HPLC–DAD analysis.

Cynarin content also varied among genotypes, showing a pattern broadly consistent with total phenolic content. Luteolin-7-O-glucoside exhibited considerable variation, indicating differences in flavonoid accumulation among genotypes. In contrast, rhoifolin showed relatively minor variation, suggesting a more stable accumulation pattern across the evaluated genotypes. Syringin levels also differed among genotypes, contributing to the overall phenolic profile diversity.

### Hierarchical clustering

Hierarchical cluster analysis grouped the genotypes into two main clusters based on the evaluated traits (Fig. 3). Bilecik and Taraklı formed a distinct group, separated from the remaining genotypes. The other genotypes clustered together, although sub-grouping was observed within this cluster, reflecting intermediate similarities in morphological and biochemical characteristics.

**Figure 3:**
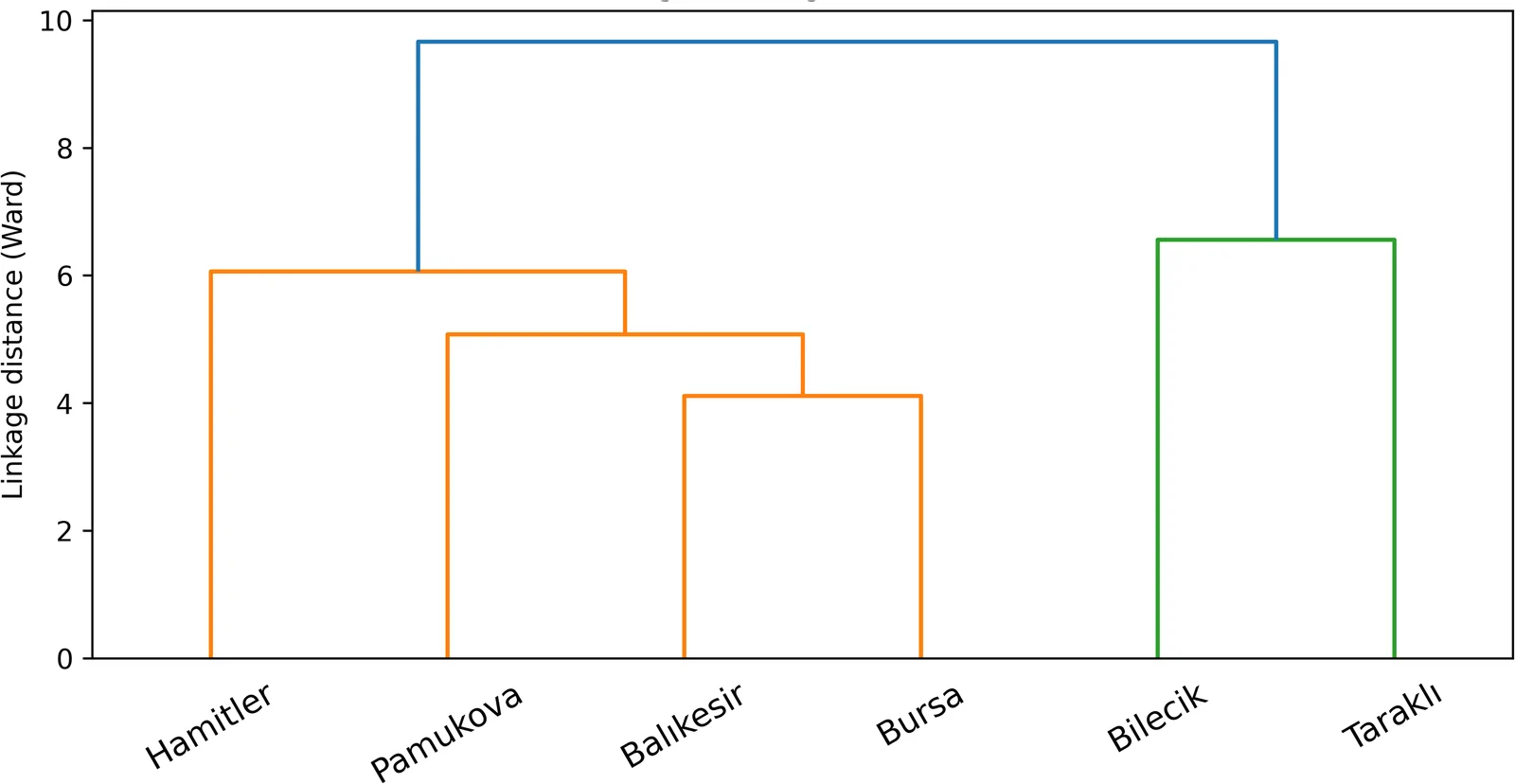
Hierarchical clustering dendrogram of globe artichoke genotypes constructed using Ward’s method based on standardized biochemical and morphological traits.

### Principal component and correlation analysis

Principal component analysis (PCA) explained 71.15% of the total variation with the first two principal components (Fig. 4). The first principal component (PC1) accounted for the majority of the variation and was mainly associated with phenolic compounds and antioxidant parameters. The second component (PC2) contributed additional variation related to morphological and pigment traits.

**Figure 4:**
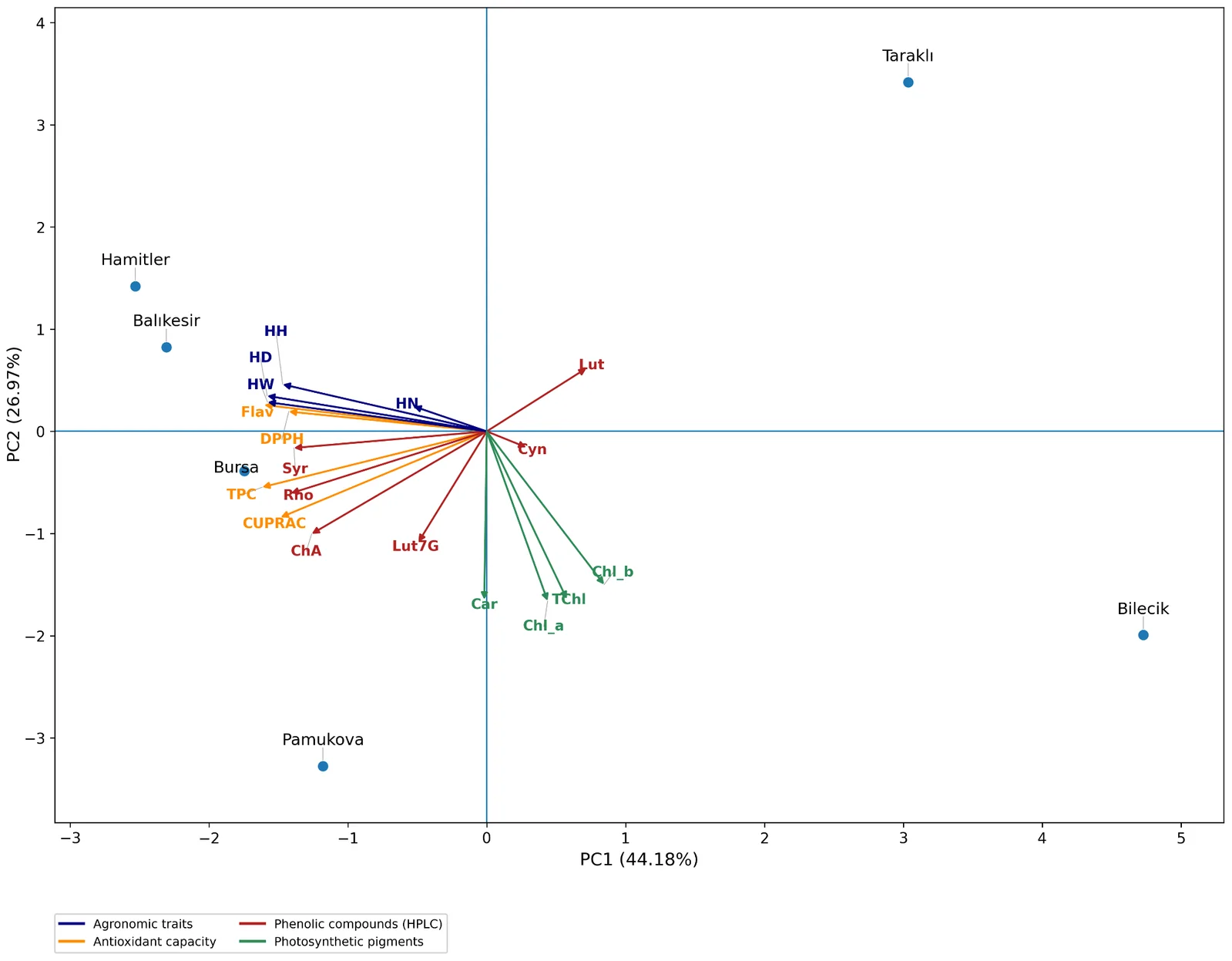
PCA biplot showing the multivariate distribution of globe artichoke genotypes based on morphological, phenolic, pigment, and antioxidant traits (PC1: 44.18%; PC2: 26.97%). Car, total carotenoids; ChA, chlorogenic acid; Chl a, chlorophyll a; Chl b, chlorophyll b; CUP, cupric reducing antioxidant capacity; Cyn, cynarin; DPPH, 2,2-diphenyl-1-picrylhydrazyl radical scavenging activity; HD, head diameter; HH, head height; HN, head number; HW, head weight; Lut, luteolin; Lut7G, luteolin-7-O-glucoside; Rho, rhoifolin; Syr, syringin; TChl, total chlorophyll; TFC, total flavonoid content; TPC, total phenolic content.

Genotypes were clearly separated along the PC1 axis, with Pamukova positioned distinctly due to its high phenolic content and antioxidant capacity, while Bilecik was characterised by its pigment-related traits. This separation indicates the strong influence of biochemical parameters on genotype differentiation.

Correlation analysis revealed strong positive relationships among total phenolic content, total flavonoids, DPPH activity, CUPRAC activity, and several individual phenolic compounds (Fig. 5). These associations indicate a close linkage between phenolic metabolism and antioxidant capacity.

**Figure 5:**
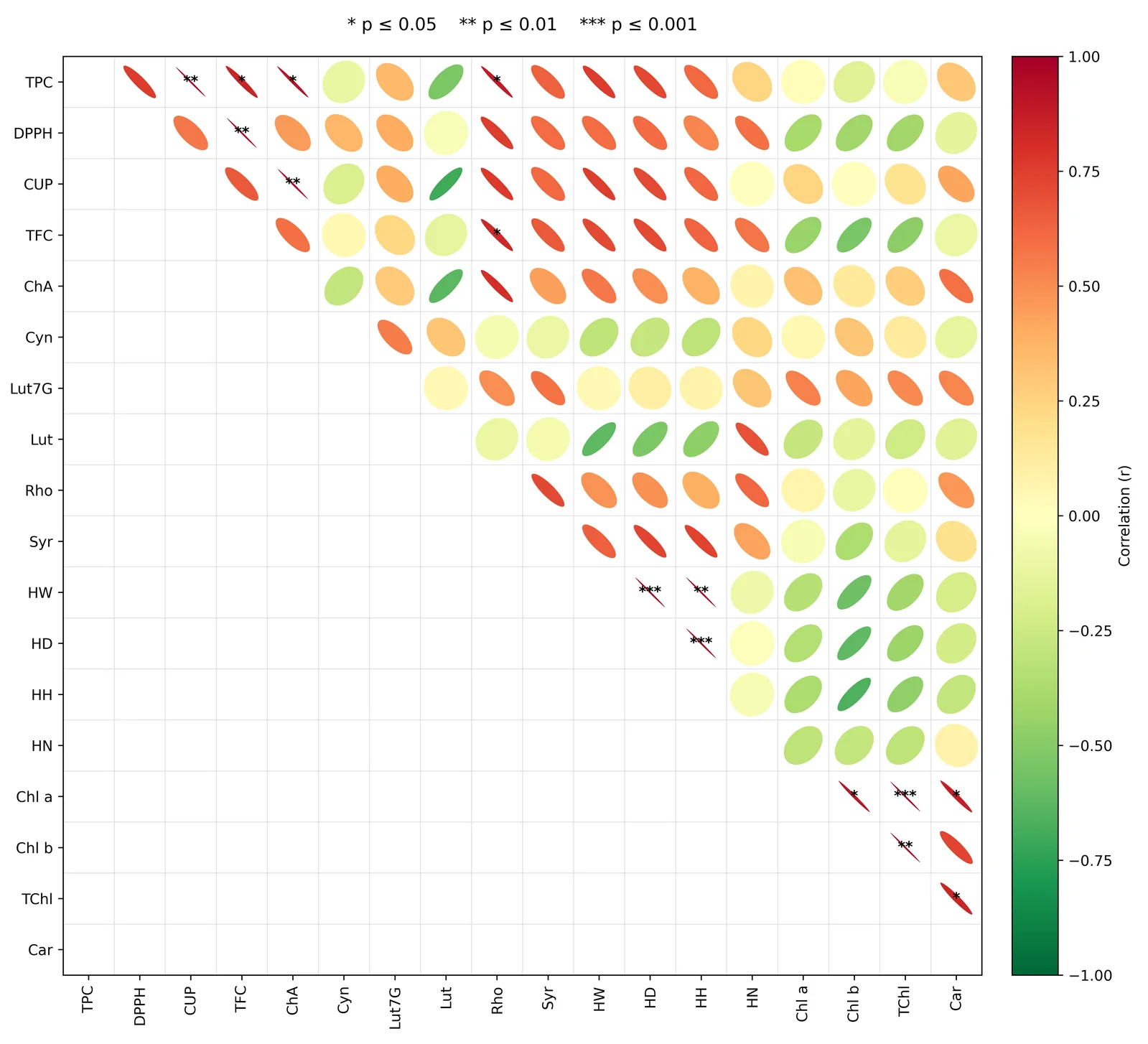
Correlation analysis among morphological traits, photosynthetic pigments, antioxidant parameters, and individual phenolic compounds in globe artichoke genotypes. Significance levels: *P ≤ 0.05, **P ≤ 0.01, ***P ≤ 0.001. Abbreviations: TPC, total phenolic content; TFC, total flavonoid content; DPPH, 2,2-diphenyl-1-picrylhydrazyl radical scavenging activity; CUP, cupric reducing antioxidant capacity; ChA, chlorogenic acid; Cyn, cynarin; Lut7G, luteolin-7-O-glucoside; Lut, luteolin; Rho, rhoifolin; Syr, syringin; HW, head weight; HD, head diameter; HH, head height; HN, head number; Chl a, chlorophyll a; Chl b, chlorophyll b; TChl, total chlorophyll; Car, total carotenoids.

In contrast, correlations between morphological traits (head weight, diameter, and height) and biochemical parameters were generally weak. This suggests that variation in yield-related traits is largely independent of variation in phenolic composition and antioxidant activity. The overall correlation pattern indicates that morphological and biochemical traits showed different patterns of variation among the evaluated globe artichoke genotypes.

## Discussion

The uniformity of soil characteristics across the experimental site indicates that within-site environmental variability was relatively limited; therefore, the observed differences among genotypes are likely to reflect genotypic variation under the specific environmental conditions of this study. This provides a useful basis for comparing genotype performance under uniform field conditions, although genotype × environment interactions could not be assessed because the experiment was conducted at a single location.

The significant variation observed in morphological traits indicates considerable phenotypic diversity among the evaluated local globe artichoke genotypes under the experimental conditions. Differences in head weight, diameter, and height may reflect genotype-related growth patterns and biomass allocation responses under the given field conditions. The higher head weight observed in Hamitler suggests that this genotype may have greater yield potential under the conditions of this study. Similar levels of morphological variability have been reported in artichoke germplasm studies, highlighting the importance of local populations as a source of variability for yield improvement (Ciancolini et al., 2012; Mauro et al., 2012). However, the relatively weak correlations observed between morphological and biochemical traits in this study suggest that high yield performance does not necessarily coincide with enhanced phytochemical quality, indicating that these trait groups may be regulated through partially independent pathways.

Variation in photosynthetic pigment composition among genotypes may indicate differences in physiological performance and photosynthetic efficiency. Higher chlorophyll content, as observed in Bilecik and Pamukova, is generally associated with increased light-harvesting capacity and potentially higher photosynthetic rates. Carotenoids, on the other hand, play a crucial role in photoprotection by scavenging reactive oxygen species and preventing oxidative damage to the photosynthetic apparatus. The relatively high carotenoid levels observed in certain genotypes may be associated with enhanced photoprotective capacity, although further multi-environment studies would be needed to confirm their role in stress tolerance. These findings are consistent with previous studies indicating that pigment accumulation is genotype-dependent and linked to physiological adaptability (Liava et al., 2021; Rau et al., 2022). Nevertheless, the lack of a direct relationship between pigment content and phenolic accumulation suggests that these traits were not strongly associated in the present dataset.

The pronounced variation in total phenolic content and antioxidant activity highlights the central role of phenolic metabolism in determining the functional quality of globe artichoke. The strong correspondence between total phenolics and antioxidant capacity confirms that phenolic compounds are the major contributors to radical scavenging activity, as reported in earlier studies (Bonasia et al., 2023; Dabbou et al., 2017). The superior performance of Pamukova in both phenolic accumulation and antioxidant activity may indicate a greater capacity for phenolic compound accumulation under the conditions of this study. This characteristic suggests that Pamukova may be a promising candidate for value-added horticultural products with enhanced health-promoting properties.

The predominance of chlorogenic acid among individual phenolic compounds is consistent with the established phytochemical profile of artichoke, where caffeoylquinic acid derivatives represent the major bioactive constituents (Liava et al., 2021; Pandino, Lombardo & Mauromicale, 2013). The higher accumulation of chlorogenic acid and cynarin in certain genotypes further supports their contribution to antioxidant capacity and nutritional quality. Variations in luteolin derivatives and other flavonoids indicate differences in flavonoid accumulation patterns among the evaluated genotypes. These differences may reflect variation in secondary metabolite profiles, although further molecular and multi-environment studies are needed to clarify the underlying regulatory mechanisms.

Multivariate analyses provided additional insights into genotype differentiation. The separation of genotypes in both hierarchical clustering and principal component analysis suggests that biochemical traits, particularly phenolic composition and antioxidant capacity, contributed substantially to the observed variation. This finding underscores the importance of including biochemical parameters in germplasm evaluation, as reliance on morphological traits alone may underestimate functional diversity. Similar patterns of genotype discrimination based on combined datasets have been reported in previous studies (Alicandri et al., 2023; Petropoulos et al., 2019).

The strong positive correlations among phenolic compounds, flavonoids, and antioxidant activity further confirm the integrated nature of phenolic metabolism. In contrast, the weak relationships between morphological and biochemical traits suggest that yield-related characteristics and secondary metabolite accumulation were not strongly associated in the present dataset. This may have implications for breeding strategies, as simultaneous improvement of yield and nutritional quality may require direct evaluation of both agronomic and biochemical traits rather than relying only on morphological traits. Comparable findings have been reported in earlier studies (Bonasia et al., 2023; Salekzamani, Ebrahimi-Mameghani & Rezazadeh, 2019).

Overall, the results indicate that the evaluated globe artichoke genotypes differed in physiological and biochemical traits under the uniform environmental conditions of this study. This highlights the potential importance of genotype selection for improving both agronomic performance and functional quality. Genotypes characterised by high phenolic content and antioxidant capacity represent valuable genetic resources for breeding programmes aimed at enhancing the nutritional value and health-promoting properties of artichoke. At the same time, high-yielding genotypes provide opportunities to improve productivity, suggesting that combining complementary traits through breeding could be an effective strategy for developing superior cultivars.

## Conclusions

The present study revealed substantial variation among genotypes in morphological traits, photosynthetic pigments, phenolic composition and antioxidant capacity among globe artichoke genotypes grown under uniform conditions. Pamukova emerged as a promising genotype with high phenolic content and antioxidant activity, whereas Bilecik exhibited superior pigment accumulation. The predominance of chlorogenic acid across genotypes highlights its key role in artichoke phytochemistry. Multivariate analyses demonstrated that biochemical traits were the main drivers of genotype differentiation. These findings provide valuable insights for germplasm evaluation and may support the selection of promising genotypes for breeding programmes aimed at enhancing both agronomic performance and functional quality. However, further multi-location studies across different altitudes and ecological conditions are needed to assess the stability, adaptability, and genotype × environment interactions of the promising genotypes identified in this study.

## Additional Information and Declarations

### Data Availability

The following information was supplied regarding data availability: The data is available at Zenodo: - SONMEZ, I. (2026). Supplementary Dataset for “Morphological Traits, Photosynthetic Pigments and Phenolic Metabolism in Globe Artichoke (*Cynara scolymus* L.) Genotypes”. Zenodo. https://doi.org/10.5281/zenodo.22200713.
